# Treatment of discospondylitis in dogs: A systematic review

**DOI:** 10.1002/vetr.70334

**Published:** 2026-01-29

**Authors:** Vasileios Ioannis Vallios, Evdokia Sourla, Daniel Low, Holger A. Volk, Marios Charalambous

**Affiliations:** ^1^ Hannover Univeristy, Clinic for Small Animals, University of Veterinary Medicine Hannover Hannover Germany; ^2^ St George's Vets IVC Wolverhampton United Kingdom; ^3^ Birmingham and Midland Eye Centre Birmingham UK; ^4^ Frank. Pet Surgeons IVC Leeds UK; ^5^ Swift Referrals IVC Wetherby UK; ^6^ Blaise Referrals IVC Birmingham UK

**Keywords:** canine, discospondylitis, diskospondylitis, treatment, vertebral endplate infection

## Abstract

**Background:**

Both medical and surgical techniques have been reported to manage canine discospondylitis, although data on clinical effectiveness and long‐term prognosis remain limited.

**Methods:**

A systematic review of studies on treatment approaches, follow‐up data and investigation methods was conducted. Each study was evaluated for evidence quality (study design, sample size, disease characterisation, healing assessment and risk of bias) and outcome measures (treatment success, follow‐up methods and long‐term outcomes) using the Cochrane risk of bias tool.

**Results:**

Forty‐eight studies on medical and surgical management were included. All were classified as low‐quality evidence with a high risk of bias. Medical and surgical interventions had success rates of 68.75% and 75%, respectively.

**Limitations:**

All studies had a high risk of bias and low‐quality evidence due to their retrospective design and lack of randomisation.

**Conclusion:**

Antibiotics should be considered the first‐line treatment when vertebral instability or disc extrusion is absent, guided by culture and sensitivity testing to ensure appropriate selection, with a mean treatment duration of 105 days. Surgical intervention is reserved for cases of failure of medical management and spinal instability, while percutaneous discectomy represents a promising adjunctive option in selected patients.

## BACKGROUND

Discospondylitis (DS) refers to the inflammation of the vertebral endplates, with intervertebral disc involvement, due to an infectious agent. Infection most commonly occurs through haematogenous spread, although foreign bodies and iatrogenic causes have also been reported.^1^, ^2^, ^3^ Bacteria such as *Staphylococcus* species (*Staphylococcus aureus* and *Staphylococcus intermedius*), followed by *Brucella canis* and *Escherichia coli*, are identified most frequently; however, fungi and algae have also been isolated, with the most common being *Aspergillus* species.^4^, ^5^, ^6^, ^7^ DS is a disease characterised by non‐specific clinical signs, varying from mild to severe, with the most common signs being pain and pyrexia. Typically, there are no or only mild neurological deficits; however, if instability, disc extrusion, myelitis or concurrent empyema occurs, neurological deficits, such as ataxia and paresis or plegia, might also manifest.

DS is reported more frequently in dogs, while cases in other species remain rarely documented in the literature.^8^ Male dogs are twice as likely to suffer from DS compared to females, while the purebred dogs are more common than the crossbreeds.^4^, ^9^, ^10^, ^11^ Neutered and sexually intact males had similar odds of being a case.^4^ The diagnosis is made by a combination of physical and neurological examinations and imaging findings. Radiographs can be sufficient for diagnosis, but most of the time, radiographic signs cannot be seen until 2‒8 weeks after the onset of clinical signs.^11^ Consequently, advanced imaging is recommended, as it could show early‐stage DS and better evaluate the epidural space and the paravertebral soft tissues.^12^ In some cases, no radiographic signs of DS have been reported, but computed tomography (CT) and magnetic resonance imaging (MRI) reveal abnormalities compatible with DS. There is significant agreement between the two aforementioned modalities (CT and MRI).^9^ Culturing material from the intervertebral disc, blood or urine is highly recommended to confirm the diagnosis and identify the causative agent. Culture of samples from the intervertebral disc, by using either percutaneous needle aspiration guided by fluoroscopy or CT, or surgical biopsy, has the highest sensitivity and could provide a more accurate diagnosis.^13^, ^14^

Various treatment methods have been used for managing DS in dogs, including both medical and surgical methods, with antibiotics and pain relief being the most common choices.^15^ Surgical treatment is primarily used when medical treatment is unsuccessful and/or when presumed vertebral instability is present, particularly in the presence of fractures or subluxation. Addressing these issues, along with the appropriate antimicrobial treatment, can lead to the resolution of infection. The same antibiotics are used whether the management is solely medical or combined with surgery.^16^ Cephalexin and amoxycillin‒clavulanic acid have been reported to be the most commonly prescribed antibiotics, but an appropriate antibiotic might be chosen after culture and sensitivity.^9^, ^10^ In the case of fungal aetiology, ketoconazole, itraconazole and fluconazole have been successfully used.^17^, ^18^

There are three major groups of surgical treatment options: fenestration and curettage of the intervertebral disc, decompression of the spinal cord with or without stabilisation of the vertebral column and discectomy.^19^, ^20^, ^21^, ^22^ Clinical information on the effectiveness of these treatments remains limited, with most evidence derived from retrospective observational studies.^9^, ^10^, ^19^, ^23^ There are no blinded randomised clinical trials (bRCTs), non‐blinded randomised clinical trials (nbRCTs) or non‐randomised clinical trials (nRCTs) available in the literature. Instead, there are only case series and observational retrospective studies, which are considered to provide low‐quality evidence.

To the authors’ knowledge, this is the first systematic review to assess studies reporting the treatment outcomes of DS, comparing the outcomes of medical versus surgical management.

## MATERIALS AND METHODS

### Search strategy

The objective of this study was to conduct a systematic review of the literature regarding the diagnosis and treatment of canine DS. The methodology of this study was based on a previous systematic review conducted by the authors (M.C. and H.V.).^24^ To explore this, several research databases have been used to identify the studies, using a set of targeted keywords, such as discospondylitis OR diskospondylitis AND dogs OR canine OR small animal OR companion animals AND/OR surgical treatment OR surgery OR decompression OR hemilaminectomy OR ventral slot OR stabilisation OR conservative OR antibiotics OR itraconazole OR antifungal. The electronic databases included PubMed, VetSRev and Google Scholar. The search included papers published between 1 January 1980 and 31 December 2023, without imposing any language restrictions. Studies written in languages other than English were initially evaluated using translation software such as Google Translate and Deepl Translate. This process aimed to gather comprehensive information and data from studies to address the optimal approach to the treatment of DS.

### Inclusion and exclusion criteria

The evaluation of studies was conducted based on comprehensive inclusion criteria aimed at assessing and comparing medical versus surgical treatment of DS. These criteria were structured as follows:

- Criterion 1—type of study: It included peer‐reviewed studies in English or translated, published from 1 January 1980 to 31 December 2023. Experimental randomised studies, analytical cohort studies, case‒control and cross‐sectional surveys and descriptive case series and reports were accepted. Ex vivo studies and reviews were excluded.
- Criterion 2—type of case definition: It excluded cases involved dogs with DS and concurrent spinal cord diseases related or unrelated to DS, such as myelitis, abscess or central nerve system diseases. Advanced imaging (MRI and CT), surgical biopsy and culture, percutaneous fine‐needle aspiration or cerebrospinal fluid (CSF) analysis were preferable but not essential. Age and breed were not restricted. Studies lacking follow‐up data were excluded due to insufficient evidence for treatment success evaluation.
- Criterion 3—type of treatment: Dogs with DS undergoing any kind of medical or surgical treatment were included. For medical treatment, reporting of drug doses, administration frequency and treatment duration was preferable. Dogs treated with methods beyond medical or surgical interventions, such as homeopathy, acupuncture, nerve stimulators, chiropractic and food trials, were excluded.
- Criterion 4—type of outcome: Studies examining outcomes related to the severity of signalment, diagnostic tool effectiveness, treatment response and complications were included. Assessment of response required evaluation by a veterinarian, preferably involving either follow‐up appointments or telephone communication. Studies were expected to report the time taken for treatment to improve the reported clinical signs and the duration of remission or relapse.

### Study selection

A two‐stage process was implemented to identify included studies by the first author. Initially, the study planning was structured using the ‘Patient/Population Intervention, Comparison, and Outcomes (PICO) and Search Strategy Worksheet’, incorporating the databases, keywords, inclusion categories and relevant studies in the field. Initially, the review concentrated on pinpointing studies directly pertinent to the systematic review questions based on the ‘Preferred Reporting Items for Systematic Reviews and Meta‐Analyses (PRISMA) 2020 flow diagram’ (stage 1), and second, studies that provided comprehensive details for evaluating the outcome measures were selected (stage 2). In stage 1, studies were elected that fulfilled the following criteria: (a) criterion 1 (see above) and (b) studies with inaccessible abstracts or PDFs were also excluded. In this stage, the studies were evaluated only by the title, the abstract and the presence of treatment data. At stage 2, the studies were evaluated based on criteria 2, 3 and 4 by reading the full text and recording the data in an EXCEL table to assess the quality of evidence and the outcome measures. Treatment outcomes have been evaluated as described in ‘Assessment of outcome measures’ section. This systematic approach ensured a comprehensive and rigorous assessment of selected studies for inclusion in the review.

### Assessment of quality of evidence

The included studies underwent an evaluation of their evidence quality, considering various critical factors. These factors encompassed an analysis of the study design, assessment of blinding, randomisation and use of control, group size, duration of the study, disease characterisation and assessment of the risk of bias. The assessment targeted a comprehensive understanding of the reliability and robustness of the evidence presented in these studies.

### Study design

In the review process, each selected trial underwent an assessment of evidence based on its study design and classification into two groups. Group A comprised bRCTs, considering higher quality evidence, followed by nbRCTs, and then nRCTs. On the contrary, group B included uncontrolled clinical trials, cohort studies, observation studies, case series (when studies with two to five cases) and case reports (when only one case was reported), which generally provide lower quality evidence.^25^ Moreover, a three‐part system of evidence quality was utilised to assess and find the weaknesses and the strengths of each study in each group through (a) group size, (b) disease characterisation and (c) overall risk of bias based on the methodological quality. For instance, within bRCTs studies, with large group sizes, comprehensive examinations, such as advanced imaging with biopsy and culture with the diagnosis of DS, alongside reporting outcomes specifically for DS, demonstrated a low risk of bias, thus providing high‐quality evidence. Overall, studies in group A provided higher quality evidence with a lower risk of bias, compared with the studies in group B. The studies selected in stage 2 are summarised in Table 1, with the assessment of quality evidence.

**TABLE 1:** Cochrane risk bias score‐based grading for the overall risk of bias in each of the 48 reviewed studies.

Studies	Randomisation of sequence generation	Allocation concealment	Blinding participants and personnel	Blinding of outcome assessment	Incomplete outcome data	Selective reporting	Other bias	Overall risk of bias
Adamo and Cherubini^52^	3	3	3	3	2	2	3—retrospective character, antibiotics prior diagnosis	19—high risk of bias
Affenzeller et al.^1^	3	3	3	3	3	2	3—retrospective character	20—high risk of bias
Anderson and Binnigton^46^	3	3	3	3	3	2	3—retrospective character, duration of the study less than 6 months	20—high risk of bias
Auger et al.^54^	3	3	3	3	2	2	3—no specific cause, retrospective character, antibiotics prior diagnosis	19—high risk of bias
Berry and Leisewitz^29^	3	3	3	3	3	2	3—no homogeneity of the management of the cases, less than 6 months duration of the study, retrospective character	20—high risk of bias
Bree et al.^15^	3	3	3	3	3	2	3—retrospective character, infection cause not found	20—high risk of bias
Brocal et al.^42^	3	3	3	3	3	2	3—duration of study less than 6 months, retrospective study	20—high risk of bias
Canal et al.^55^	3	3	3	3	3	2	2—retrospective character	19—high risk of bias
Chen et al.^47^	3	3	3	3	3	2	3—retrospective character duration less than 6 months	20—high risk of bias
Cherubini et al.^48^	3	3	3	3	2	2	2—retrospective character, duration less than 6 months	19—high risk of bias
Cook et al.^18^	3	3	3	3	3	2	3—retrospective character, duration less than 6 months, study does not focus on discospondylitis, treatment protocol changed due to financial reasons	20—high risk of bias
Corrente et al.^56^	3	3	3	3	3	2	3—retrospective character, duration less than 6 months	20—high risk of bias
De Freitas et al.^27^	3	3	3	1	2	2	2—retrospective character	16—moderate/high risk of bias
Denny et al.^28^	3	3	3	3	3	2	2—retrospective character	19—high risk of bias
Devecioğlu et al.^30^	3	3	3	3	3	2	3—retrospective character, duration less than 6 months	20—high risk of bias
Escauriaza et al.^49^	3	3	3	3	3	2	3—retrospective character, duration less than 6 months	20—high risk of bias
Finnen et al.^3^	3	3	3	3	1	2	3—retrospective character, no homogeneity of the management of the cases	18—moderate/high risk of bias
Forbes et al.^50^	3	3	3	3	2	2	3—retrospective character, duration less than 6 months	19—high risk of bias
Golini et al.^44^	3	3	3	3	3	2	3—retrospective character, duration less than 6 months, letter to the editor	20—high risk of bias
Graham et al.^41^	3	3	3	3	3	2	3—retrospective character, duration less than 6 months, no homogeneity of the management of the cases	20—high risk of bias
Hugnet et al.^32^	3	3	3	3	3	2	3—retrospective character, duration less than 6 months	20—high risk of bias
James et al.^53^	3	3	3	3	2	2	3—retrospective character, antibiotics prior diagnosis	19—high risk of bias
Jeong et al.^51^	3	3	3	3	3	2	3—retrospective character, duration less than 6 months	20—high risk of bias
Kawalilak et al.^6^	3	3	3	3	3	2	3—retrospective character, conflict of interest, financial support	20—high risk of bias
Kelly et al.^5^	3	3	3	3	3	2	3—retrospective character, duration less than 6 months, financial support	20—high risk of bias
Kerwin et al.^36^	3	3	3	3	3	2	2—retrospective character	19—high risk of bias
Kinzel et al.^14^	3	3	3	3	1	2	2—retrospective character, duration less than 6 months	18—moderate/high risk of bias
Kornegay and Barber^19^	3	3	3	3	3	2	2—retrospective character	19—high risk of bias
Kuşçu et al.^45^	3	3	3	3	3	2	3—letter to the editor, duration of the study less than 6 months, retrospective character	20—high risk of bias
Lobetti^37^	3	3	3	3	2	2	3—retrospective character, duration less than 6 months	19—high risk of bias
MacFarlane and Iff^2^	3	3	3	3	3	2	3—retrospective character, duration less than 6 months, letter to the editor	20—high risk of bias
Manino et al.^7^	3	3	3	3	3	2	3—retrospective character, duration less than 6 months	20—high risk of bias
Manou et al.^21^	3	3	3	3	1	2	2—retrospective character	17—moderate/high risk of bias
McKee et al.^16^	3	3	3	3	3	2	2—retrospective character	19—high risk of bias
Nye et al.^35^	3	3	3	3	3	2	3—retrospective character, no homogeneity in the management of the cases	20—high risk of bias
Pacurar et al.^31^	3	3	3	3	3	2	3—retrospective character, duration less than 6 months	20—high risk of bias
Pilkilngton et al.^10^	3	3	3	3	3	2	3—retrospective character, no homogeneity of the management of the cases	20—high risk of bias
Polak et al.^57^	3	3	3	3	3	2	2—retrospective character	19—high risk of bias
Renwick et al.^40^	3	3	3	3	1	2	2—retrospective character	17—moderate/high risk of bias
Rizzo et al.^38^	3	3	3	3	3	2	2—retrospective character	19—high risk of bias
Royaux and Guilherme^22^	3	3	3	3	1	2	2—retrospective character	17—moderate/high risk of bias
Seo et al.^43^	3	3	3	3	3	2	2—retrospective character	19—high risk of bias
Shamir et al.^34^	3	3	3	1	3	2	3—retrospective character, included only dog with successful treatment	17—moderate/high risk of bias
Siems et al.^58^	3	3	3	3	3	2	3—retrospective character, no identification of agent	20—high risk of bias
Trub et al.^23^	3	3	3	3	3	2	3—retrospective character, no homogeneity of the management of the cases, financial support	20—high risk of bias
Van Hoof et al.^9^	3	3	3	3	3	2	3—retrospective character, no homogeneity of the management of the cases	20—high risk of bias
Zanatta et al.^39^	3	3	3	3	3	2	2—retrospective character	19—high risk of bias
Zhang et al.^33^	3	3	3	3	3	2	3—retrospective character, duration less than 6 months	20—high risk of bias

### Study group size

Based on the studies' respective group sizes, they classified in the following groups: ‘good’, if they involved more than 50 dogs, ‘moderate’ when 20‒49 dogs, ‘small’ for studies with 10‒19 dogs and ‘very small’ when less than 10 dogs were involved.

### Subject enrolment quality—disease characterisation

Disease characterisation is established through investigations, classifying the cases based on their effectiveness in evaluating subject enrolment:

- ‘Well characterised’: patients underwent clinical and neurological examination AND/OR radiographs AND blood examinations and/or culture OR urine analysis and culture AND diagnosis was confirmed by advanced imaging such as MRI or CT AND/OR surgical biopsy and culture OR percutaneous fine‐needle aspiration of the affected disc (CT or ultrasound guided). CSF analysis and culture were optional but recommended, as they aided in excluding concurrent conditions such as meningomyelitis.
- ‘Fairly characterised’: patient underwent clinical and neurological examination AND/OR radiographs AND blood examinations and/or culture OR urine analysis and culture AND DS was confirmed by advanced imaging, such as MRI or CT.
- ‘Poorly characterised’: patients underwent clinical and neurological examination AND radiographs AND blood examinations, and/or culture, or urine analysis and culture.
- ‘Unclearly characterised’: diagnosis only after the clinical and neurological examination (i.e., empirical diagnosis) or no or indistinct information is given about the diagnosis of DS.

#### Assessment of methodological quality

The risk of bias was evaluated by the first author, using the Cochrane ‘risk of bias’ methodology—‘Handbook for Systemic Reviews of Interventions tool’,^26^ which classified studies’ components into selection bias (sequence generation, allocation concealment), performance bias (blinding of participants or personnel), attrition bias (incomplete outcome data), reporting bias (selective reporting) and other potential risk of bias such as conflicts of interest, duration of the study less than 6 months, etc. Each component was characterised as ‘high’, ‘moderate’, ‘unclear’ or ‘low’ risk of bias by receiving a numerical score as below (scale: ‘high’ risk of bias = 3, ‘moderate’ risk of bias or ‘unclear’ = 2, and ‘low’ risk of bias = 1), culminating in a total score that indicates the overall estimated risk of bias:

- Score: 7‒9: overall ‘low’ risk of bias
- Score 10‒12: overall ‘low/moderate’ risk of bias
- Score 13‒15: overall ‘moderate’ risk of bias
- Score 16‒18: overall ‘moderate/high’ risk of bias
- Score 19‒21: overall ‘high’ risk of bias

#### Assessment of outcome measures

The assessment of outcome measures evaluated the treatment effectiveness in dogs with DS of both medical and surgical treatment. The parameters considered for outcome evaluation encompass the treatment option, the presence of relapse and the time after the application of the treatment, the investigation methods at the follow‐up, the resolution or not of DS and the time of resolution if it occurred. These factors must be addressed in the included studies. One standardised scale was utilised for each outcome criterion to ensure uniformity and facilitate appropriate comparisons between the results of the studies. This approach aimed to simplify and ease the process of comparing findings across different studies.

### Use of treatment‒relapse‒healing scale (subjective)

The studies were categorised into groups based on the treatment that was used. Studies with medical management were classified as group 1, while those where surgery took place were categorised under group 2. Dogs that were euthanased without attempting any treatment were categorised into group 3. Studies where both options were used were added to both groups. Conservative treatment includes all forms of medical treatment, while surgery included all the interventions that need general anaesthesia, opening the skin and removing or repairing a damaged anatomical structure.

All studies included follow‐up appointments after the treatment. Studies, where the dogs had a follow‐up after successful treatment in the subsequent year (>12 months), were considered to have long‐term resolution. Studies with follow‐up after successful treatment within 6‒12 months were characterised as achieving mid‐term resolution, whereas studies reported follow‐up in less than 6 months after treatment characterised short‐term resolution. To qualify a study that achieved successful treatment, it must be in at least 50% of dogs that it was applied.

The methodology the studies used to confirm the healing of the DS were categorised using the following scale, where a higher number indicates poorer methodology:

- Healing scale 1: advanced imaging (MRI or CT) or surgical biopsy with or without CSF analysis, alongside neurological examination and grading of neurological signs.
- Healing scale 2: CSF analysis and neurological examination and/or X‐rays or urine or blood culture.
- Healing scale 3: X‐rays and neurological examination and/or blood or urine examinations and culture.
- Healing scale 4: neurological examination, blood and urine examination and culture.
- Healing scale 5: follow‐up by in‐person neurological examination.
- Healing scale 6: neurological evaluation after telephonic follow‐up or when it is unclear.

### Clinical signs severity before and after treatment (objective)

The neurological examination was considered crucial to assess the neurological status of the dogs. For the purposes of this study, the following grading scale was applied. Grading before and after the treatment is important to evaluate the effectiveness of the treatment and the improvement of the patient's clinical signs. The following scale was used to grade the neurological signs of the dogs:

- Grade 0: normal.
- Grade I: painful neck/back without neurological deficits.
- Grade II: ambulatory paraparesis/tetraparesis with or without pain.
- Grade III: non‐ambulatory paraparesis/tetraparesis with voluntary movement in the affected limbs or tail.
- Grade IV: paraplegia/tetraplegia with intact deep pain perception and loss of urinary function.
- Grade V: paraplegia/tetraplegia with loss of deep pain perception and loss of urinary function.

## RESULTS

### Description of the studies

The search strategy identified 291 publications, sourced from electronic searches of PubMed, VetSRev and Google Scholar, as well as manual searches of the reference lists. Eighty‐four studies met stage 1 screening criteria. Of these, 48 studies met the stage 2 requirements and were selected for review.

All the studies were classified in group B. The study design included 29 case reports, eight case series and 11 retrospective cohort studies. All were written originally in English. Overall, 32 studies reported on medical treatment and 16 reported on surgical treatment. Five studies^9^, ^23^, ^27^, ^28^, ^29^ reported both treatments and were included in both groups 1 and 2. In the remaining five studies,^29^, ^30^, ^31^, ^32^, ^33^ the dogs were euthanased without attempting any treatment. One of these studies^29^ was a case series, and one of the patients underwent surgery, so the study was included in both groups 2 and 3.

### Disease characterisation

According to the described classification system for subject enrolment quality, 20 studies enroled well‐characterised subjects, 13 enroled fairly characterised subjects and 14 enroled poorly characterised subjects. In one study, the diagnostic procedures for enrolment of cases with DS were unclear.^18^

### Study group size

All 48 studies reported the total number of dogs (*n* = 699) evaluated (mean = 14, range 1‒386). Two of the selected studies evaluated a good number of dogs,^9^, ^10^ while two retrospective observational studies evaluated a moderate number of dogs.^19^, ^27^ Five studies evaluated a small group of patients,^14^, ^23^, ^28^, ^34^, ^35^, ^36^ while the remaining 39 studies assessed a very small number.

### Signalment and baseline characteristics of study subjects

Characteristics data, such as breed, sex, causative agent and the presence of co‐morbidity, for the enroled dogs were reported across the included studies. The most commonly reported breed was the German Shepherd dog. It was one of the most frequently represented breed in case reports,^2^, ^5^, ^15^, ^18^, ^21^, ^29^, ^32^, ^33^, ^37^, ^38^, ^39^ and one retrospective observational study^19^ accounted for 10.71% of the sample population (75/699; 95% confidence interval [CI]: 8.42%–13.01%). Furthermore, the Labrador Retriever comprised 12.43% of the sample population (87/699; 95% CI: 9.98%–14.87%) and was the most frequently reported breed in a large study of 386 dogs.^9^ Other significantly affected breeds included crossbreeds and large pure breeds, such as Boxers, Collies, Rottweilers, Dobermans and Great Danes. Medium‐sized breeds were also reported, including French Bulldogs, Spaniels, Pugs and American Staffordshire Terrier. Most studies indicated that males (60.86%; 95% CI: 57.24%–64.47%) were more affected than females.^9^, ^10^, ^19^, ^36^

The most common Gram‐negative identified bacteria were *B. canis*, *E. coli*, *Proteus* spp. and *Pseudomonas* spp. Conversely, larger studies indicated a high prevalence of Gram‐positive bacteria, such as *Staphylococcus* spp. and *Streptococcus* spp.^10^, ^14^, ^19^ Fungi and algae were also noted.^7^ In most of the studies, the patients presented with a co‐morbidity (28/48), with the most common being orthopaedic and urinary tract concurrent conditions.

### Methodological quality of included studies

According to the design criteria, all the studies were classified in group B, as no clinical trials were found in the literature. Thus, most of the studies were assessed as having a high risk of bias, resulting in a low quality of evidence outcome. Seven studies^3^, ^14^, ^21^, ^22^, ^27^, ^34^, ^40^ were considered to have a moderate/high overall risk of bias. Summaries of the risk of bias for each study are provided in Table 1.

### Risk of bias assessment (methodological quality)

No randomisation and blinding were used, as there were only studies classified in group B. Assessing selective reporting was challenging because study protocols were not reviewed beyond the published information, and no studies were assessed for this bias and thought to be free of it. Five studies presented outcomes from all included dogs, in which there were no losses between enrolment and evaluation.^3^, ^14^, ^21^, ^22^, ^40^ The same studies were considered at moderate/high or high risk of bias. In the remaining studies, some dogs were either euthanased or lost at the follow‐up or the long‐term outcome of the treatment was not evaluated, recording important aspects, such as the diagnostic approach, the clinical status and the time after treatment (Table 1).

### Acknowledgement of other sources of bias

Three studies reported financial support^5^, ^6^, ^23^ and all of them were characterised as providing a high risk of bias. In three studies, there were clear declaration of no financial support.^27^, ^35^, ^41^ In the remaining 42 studies, no financial statement was provided. No conflict of interest was clearly stated in 10 studies.^1^, ^9^, ^18^, ^21^, ^23^, ^27^, ^35^, ^41^, ^42^, ^43^ One study declared a conflict of interest.^6^

All the included studies had retrospective character, while in three of them, only the peer‐review letter had been found.^2^, ^44^, ^45^ The duration of 22 studies was less than 6 months, which was considered to be inadequate.^2^, ^5^, ^7^, ^14^, ^18^, ^29^, ^30^, ^31^, ^32^, ^33^, ^37^, ^41^, ^42^, ^44^, ^45^, ^46^, ^47^, ^48^, ^49^, ^50^, ^51^ In seven studies, there was heterogeneity in the diagnosis and treatment; thus, the disease characterisation and the outcome of the treatment were challenging to evaluate appropriately.^3^, ^9^, ^10^, ^23^, ^29^, ^35^, ^41^ In three studies, the dog was treated initially with antibiotics, before the identification of the causative agent took place, which could lead to false‐negative results of the culture and sensitivity.^52^, ^53^, ^54^ Indeed, in one of these studies, the causative agent was identified in only one of the four involved dogs.^54^ In one case report, the treatment plan was changed due to financial reasons,^18^ while one study included only dogs that were considered to be successfully treated^34^ (Table 1).

### Treatment

#### Medical management

In group 1, 32 studies were included^1^, ^2^, ^3^, ^5^, ^6^, ^7^, ^9^, ^10^, ^14^, ^15^, ^18^, ^19^, ^23^, ^27^, ^28^, ^34^, ^35^, ^36^, ^37^, ^38^, ^39^, ^41^, ^43^, ^44^, ^45^, ^46^, ^48^, ^49^, ^50^, ^51^, ^55^, ^58^ (Table 2). The primary treatments consisted of antibiotics and antifungal agents. Other supplementary drugs were also used, such as non‐steroidal anti‐inflammatories and corticosteroids. Resolution of DS was reported in 22 studies (68.75%), while treatment failure occurred in 10 studies (31.25%). In eight out of these 10 studies, the dogs either did not survive or were euthanased, whereas in the remaining two studies,^1^, ^43^ the dogs survived but experienced neurological deterioration. Of the eight studies reporting non‐survival, seven described neurological deterioration within the first 6 months, and one study reported deterioration between 6 and 12 months.^6^ In the two studies where the dogs survived but relapse occurred, recurrence was documented between 6 and 12 months.^1^, ^43^ Overall, there is fair evidence indicating that poor prognosis is associated with fungal infection.

**TABLE 2:** Study design, group size, risk of bias, disease characterisation, type of medication, healing scale, outcome and resolution of cases in which medical treatment was performed without surgical intervention.

References	Study design	Group size	Risk of bias	Disease characterisation	Type of medication	Healing scale	Outcome	Resolution
Affenzeller et al.^1^	Case series	Very small	High	Fairly	Antibiotics, painkillers, anti‐inflammatory	5	Alive	No
Anderson and Binnignton^46^	Case report	Very small	High	Poor	Antibiotics	6	Alive	Yes—mid term
Bree et al.^15^	Case report	Very small	High	Fairly	Antibiotics, steroids, painkiller	2	Alive	Yes—mid term
Canal et al.^55^	Retrospective cohort study	Very small	High	Fairly	Antibiotics	3	Alive	Yes—mid term
Cherubini et al.^48^	Case report	Very small	High	Well	Antibiotics, painkiller, anti‐inflammatory	6	Alive	Yes—short term
Cook et al.^18^	Case report	Very small	High	Unclear	Antifungal and steroids	5	Alive	Yes—mid term
De Freitas et al.^27^	Cross‐sectional retrospective study	Moderate	Moderate/high	Well	Antibiotics and painkiller	4	Alive	Yes—mid term
Denny et al.^28^	Retrospective cohort study	Small	High	Poor	Antibiotics	3	Alive, (1/9) died	Yes—long term
Escauriaza et al.^49^	Case report	Very small	High	Fairly	Antibiotics	N/A	Died	No
Finnen et al.^3^	Case series	Very small	Moderate/high	Fairly	Antibiotics, corticosteroids, painkiller	3	Alive	Yes—long term
Forbes et al.^50^	Case report	Very small	High	Fairly	Antibiotics	3	Alive	Yes—short term
Golini et al.^44^	Case report	Very small	High	Fairly	Antibiotics	3	Alive	Yes—mid term
Graham et al.^41^	Case series	Very small	High	Fairly	Antibiotics	6	Alive	Yes—mid term
Jeong et al.^51^	Case report	Very small	High	Poor	Antibiotics	4	Alive	Yes—short term
Kawalilak et al.^6^	Case report	Very small	High	Poor	Antibiotics, antifungal, painkillers	N/A	Died	No
Kelly et al.^5^	Case series	Very small	High	Poor	Antifungal	N/A	Died	No
Kerwin et al.^36^	Retrospective cohort study	Small	High	Poor	Antibiotics	4	Alive	Yes—short term
Kinzel et al.^14^	Retrospective cohort study	Small	Moderate/high	Well	Antibiotics	5	Alive	Yes—short term
Kornegay and Barber^19^	Retrospective cohort study	Moderate	High	Poor	Antibiotics	5	Alive	Yes—long term
Kuşçu et al.^45^	Case report	Very small	High	Poor	Antibiotics	4	Alive	Yes—short term
Lobetti^37^	Case report	Very small	High	Poor	Antibiotics	N/A	Died	No
Mac Farlane and Iff^2^	Case report	Very small	High	Well	Antibiotics, painkiller	N/A	Died	No
Manino et al.^7^	Case report	Very small	High	Poor	Antibiotics, painkiller	N/A	Died	No
Nye et al.^35^	Retrospective cohort study	Small	High	Well	Antibiotics	N/A	Alive (1/8 died)	Yes—short term
Pilkington et al.^10^	Retrospective case‒control study	Good	High	Well	Antibiotics	6	Alive	Yes—short term
Rizzo et al.^38^	Case report	Very small	High	Well	Antibiotics, antifungal	N/A	Died	No
Seo et al.^43^	Case report	Very small	High	Fairly	Antibiotics	3	Alive	No
Shamir et al.^34^	Retrospective/prospective cohort study	Small	Moderate/high	Fairly	Antibiotics	3	Alive	Yes—long term
Siems et al.^58^	Case report	Very small	High	Poor	Antibiotics	3	Alive	Yes—short term
Trub et al.^23^	Retrospective cohort study	Small	High	Fairly	Antibiotics	5	Alive	Yes—short term
Van Hoof et al.^9^	Retrospective case‒control study	Good	High	Fairly	Antibiotics	6	Alive	Yes—long term
Zanatta et al.^39^	Case report	Very small	High	Poor	Antibiotics, antifungal	N/A	Died	No

Abbreviation: N/A, not available.

#### Antibiotics

In 30 studies,^1^, ^2^, ^3^, ^6^, ^9^, ^10^, ^14^, ^15^, ^19^, ^23^, ^27^, ^28^, ^34^, ^35^, ^36^, ^37^, ^38^, ^39^, ^41^, ^43^, ^44^, ^45^, ^46^, ^48^, ^49^, ^50^, ^51^, ^55^, ^58^ antibiotics were administered for treatment of DS. In 20 of these studies, antibiotics were used as the sole therapeutic intervention, whereas in the remaining studies, antibiotics were combined with analgesics, corticosteroids or antifungal drugs. Altogether, 530 dogs were assessed for the long‐term outcome of antimicrobial treatment. Four studies were judged to have moderate/high risk of bias,^3^, ^27^, ^34^ while the remaining studies exhibited a high risk of bias. Disease characterisation was considered fair in 12 studies, poor in 11 and well defined in only seven studies.

Successful outcome was reported in 21 studies, with a long‐term resolution achieved in five studies,^3^, ^9^, ^19^, ^28^, ^34^ mid‐term resolution in six studies^15^, ^27^, ^41^, ^44^, ^46^, ^55^ and short‐term resolution in 10 studies.^10^, ^14^, ^23^, ^35^, ^36^, ^45^, ^48^, ^50^, ^51^, ^58^ In two studies, no resolution was achieved, but the affected dogs survived.^1^, ^43^ In the remaining seven studies, the dogs either died or were euthanased following unsuccessful antibiotic therapy. Despite this, the majority of dogs demonstrated favourable responses to antibiotic treatment.

The most frequently prescribed antibiotics were amoxicillin‒clavulanic acid (25.71%; 95% CI: 22.48%–28.95%), cephalexin (29.86%; 95% CI: 26.47%–33.25%) and enrofloxacin (9.00%; 95% CI: 6.88%–11.12%). Doxycycline was reported in six studies, predominantly case reports, with *B. canis* being identified as the causative agent. Most studies used a single antibiotic as monotherapy, with one report noting that 76 out of 112 dogs treated with a single agent achieved short‐term resolution.^10^ Ten studies employed dual antibiotics, while two reported the use of three antibiotics. Four studies did not specify the type of antibiotic used, while one study did not specify the duration.^14^ The medical treatment duration ranged from 14 to 420 days, with a mean treatment duration of 105 days. Notably, only five studies reported antibiotic selection based on sensitivity testing, all of which reported the resolution of DS.^3^, ^14^, ^19^, ^48^, ^55^ Collectively, these findings provide strong evidence that antibiotic therapy, particularly when guided by culture and sensitivity testing and combined with analgesics or anti‐inflammatory agents, should be considered the cornerstone of medical management in dogs with DS, especially in the absence of severe neurological deficits.

#### Antifungal

Five studies^5^, ^6^, ^18^, ^38^, ^39^ evaluated the effectiveness of antifungal medication in dogs with DS, with a combined sample size of six dogs. In all studies, the agent was identified via culture from urine, blood or spleen or after surgical biopsy from the affected spinal region, except for two studies,^32^, ^33^ where the pathogen was only identified postmortem. Antifungal therapy was used as a monotherapy in one single study,^5^ which resulted in treatment failure and death. In contrast, only one report documented successful treatment, in which the dog survived following a regimen that combined corticosteroids with posaconazole and itraconazole.^18^ Itraconazole was administered in four cases, while posaconazole and fluconazole were each reported once. Three studies described the concurrent use of antibiotics alongside antifungal agents.^6^, ^38^, ^39^ All studies were assessed as high risk of bias. Overall, there is suspicion of poor prognosis of DS with fungal infection; however, the available data are of very low quality of evidence, with high risk of bias, and do not allow reliable conclusions regarding the long‐term efficacy of antifungal therapy.

#### Other medications (used as secondary in the treatment plan)

Nine studies^1^, ^2^, ^3^, ^6^, ^7^, ^15^, ^18^, ^27^, ^48^ reported the use of adjunctive medications, including corticosteroids and anti‐inflammatory agents, in addition to antimicrobial therapy. The most frequently administered drugs were dexamethasone, prednisolone, gabapentin, carprofen and meloxicam. In four studies,^1^, ^2^, ^6^, ^7^ no resolution was achieved, and none of the affected dogs exhibited severe neurological deficits beyond spinal hyperesthaesia. In contrast, the remaining studies documented resolution when adjunctive medications were combined with antimicrobial treatment, even in cases with more pronounced neurological deficits than those reported in the non‐survival studies, with the exception of one study.^27^ Nevertheless, adjunctive medication may be considered a valuable addition to antimicrobial protocols, as it can improve quality of life during the treatment period.

### Surgical intervention

Sixteen studies were included in group 2,^14^, ^16^, ^21^, ^22^, ^40^, ^42^, ^47^, ^52^, ^53^, ^54^, ^56^, ^57^ while five studies overlapped between groups 1 and 2^19^, ^23^, ^27^, ^28^, ^29^ (Table 3). The reported surgical techniques included curettage and fenestration of the intervertebral disc,^19^, ^29^, ^47^, ^57^ discectomy^14^, ^22^, ^40^ and decompression with or without distraction and stabilisation.^16^, ^21^, ^27^, ^28^, ^42^, ^52^, ^53^, ^54^, ^56^ One study did not provide details of the surgical method used.^23^ Postoperatively, antibiotics and non‐steroid anti‐inflammatories were routinely prescribed. The most commonly prescribed antibiotics were amoxicillin‒clavulanic acid and cephalexin, although ciprofloxacin, enrofloxacin, marbofloxacin, clindamycin, streptomycin, erythromycin, doxycycline, erythromycin and rimphabycin were also reported. Voriconazole was prescribed in one case following vertebral stabilisation with a biplanar external fixator.^42^ Neurological deficits were present in nearly all surgical cases, ranging from mild paresis to plegia. Most plegic patients retained deep pain sensation, with the exception of one study that included three dogs, all of which achieved resolution following surgery.^52^ In another study, neurological status was less clearly described, with ‘plegia’ reported without specification of deep pain sensation; in this study, only 10% of surgically managed dogs recovered.^28^ Antibiotic selection was guided by sensitivity testing in only three studies, all of which reported successful outcomes.^14^, ^16^, ^22^, ^52^ The duration of postoperative antimicrobial treatment ranged from 14 to 750 days, with a median of 45.5 days and a mean of approximately 154 days. Resolution of DS was not achieved in four studies.^29^, ^42^, ^56^, ^57^ In three of these, the causative pathogen was fungal, while in the remaining study, the aetiological agent was *B. canis*. Three out of four studies showed a relapse within the first 6 months after surgery, while in the remaining case,^56^ no time was reported. In seven studies, the dogs showed long‐term (>1 year) resolution of DS.^16^, ^19^, ^21^, ^22^, ^28^, ^53^, ^54^ The success rate of surgical intervention was approximately 75%, highlighting its role as a valuable treatment option, particularly when guided by culture and sensitivity results. Additionally, surgery can facilitate accurate antibiotic selection through culture and sensitivity testing of the affected area, allow decompression of an empyema or extruded disc and provide spinal stabilisation when required.

**TABLE 3:** Study design, sample size, risk of bias, disease characterisation, type of surgical method and medications used, healing scale, outcomes, resolution status and corresponding time to resolution in all studies involving surgical interventions (group 2).

References	Study design	Group size	Risk of bias	Disease characterisation	Type of surgery/medication	Healing scale	Outcome	Resolution
Adamo and Cherubini^52^	Case series	Very small	High	Well	Decompression and curettage, antibiotics after C and S tests, anti‐inflammatory, vitamin B	3	Alive	Yes—short term
Auger et al.^54^	Case series	Very small	High	Well	Laminectomy and stabilisation with Ex‐Fix, antibiotics and anti‐inflammatory	3	Alive	Yes—long term
Berry and Leisewitz^29^ ^,^ a	Case series	Very small	High	Well	Curettage	N/A	Died	No
Brocal et al.^42^	Case report	Very small	High	Well	Stabilisation with Ex‐Fix, Voconazole	N/A	Died	No
Chen et al.^47^	Case report	Very small	High	Well	Curettage	6	Alive	Yes—mid term
Corrente et al.^56^	Case report	Very small	High	Well	Decompression, streptomycin	N/A	Died	No
De Freitas et al.^27^ ^,^ b	Cross‐sectional retrospective study	Very small (only five surgical)	High	Well	Decompression, antibiotics, painkillers	1	Alive (33% died, 66% alive)	Yes—mid term
Denny et al.^28^ ^,^ b	Retrospective cohort study	Very small (10% surgical)	High	Poor	Decompression and stabilisation (fusion with a screw)	3	Alive	Yes—long term
James et al.^53^	Case report	Very small	High	Well	Decompression, antibiotics	4	Alive	Yes—long term
Kornegay and Barber^19^ ^,^ b	Retrospective cohort study	Small (surgical, *N* = 10)	High	Poor	Curettage, antibiotics	5	Alive	Yes—long term
Manou et al.^21^	Case report	Very small	Moderate/high	Well	Stabilisation (ventral slot approach of LS) PMMA and omentalisation, antibiotics, anti‐inflammatory, painkillers	3	Alive	Yes—long term
McKee et al.^16^	Case series	Very small	High	Well	Distraction and stabilisation using two pins—cross‐technique and bone graft, antibiotics after culture, painkillers	3	Alive	Yes—long term
Polak et al.^57^	Case report	Very small	High	Well	Fenestration, curettage, antibiotics	N/A	Died	No
Renwick et al.^40^	Case report	Very small	Moderate/high	Fairly	Discectomy and stabilisation (annuloectomy) using 3.5 mm pedicle screws and PMMA, antibiotics and painkillers	3	Alive	Yes—mid term
Royaux and Guilherme^22^	Case report	Very small	Moderate/high	Well	Discectomy and stabilisation using pedicle screws 3.5 mm, transarticular pin and PMMA, antibiotics after culture	3	Alive	Yes—long term
Trub et al.^23^ ^,^ b	Retrospective cohort study	Very small (3/18)	High	Fairly	N/A	5	Alive	Yes—short term

Abbreviations: C, culture; Ex‐Fix, external fixation; LS, lumbosacral; PMMA, polymethylmethacrylate; S, sensitivity.

^a^ This study was also included in group 3 (Table 4), as the remaining dog was euthanased without attempting any treatment.

^b^ These studies were also allocated in group 1 (Table 2), as the majority of the included dogs were treated only medically.

### Curettage and fenestration

Four studies reported curettage and fenestration of the affected disc.^19^, ^29^, ^47^, ^57^ Three of these were case reports,^29^, ^47^, ^57^ with survival reported in only one.^47^ Both studies provided well disease characterisation but were classified as having a high risk of bias. The fourth study was a retrospective observational study involving 32 dogs, of which only 10 dogs underwent surgical intervention.^19^ Six patients received curettage alone, while the remaining four underwent curettage combined with either hemilaminectomy or stabilisation. All surgically treated patients showed improvement and eventual resolution. However, this study showed a high risk of bias, a poor definition of the disease, and inadequate outcome assessment methodology, reflected by a high healing scale. Collectively, the available evidence for curettage and fenestration as standalone techniques is limited, and these procedures should not be considered first‐line surgical options.

### Decompression and/or stabilisation

Eleven studies described decompressive techniques (hemilaminectomy, laminectomy or ventral slot)^27^, ^52^, ^53^, ^56^ or distraction and stabilisation^16^, ^21^, ^42^ or combined procedures.^28,54^ In two studies, stabilisation was performed alongside discectomy.^22^, ^40^ A total of 32 patients were treated with decompression and/or stabilisation, with only two failing to recover or survive. The remainder achieved resolution, categorised as short term,^52^ mid term^27^, ^40^ or long term.^16^, ^21^, ^22^, ^28^, ^53^, ^54^ Most studies involved a very small group of dogs and were classified as high risk of bias, with the exception of one,^21^ which was considered moderate‐to‐high risk. Reported stabilisation techniques varied, including external fixation following laminectomy,^42^, ^54^ polymethylmethacrylate (PMMA) with omentalisation of lumbosacral via ventral slot approach,^21^ two pins with cross‐pin technique,^16^ pedicle screw, PMMA and annulectomy^40^ and combined pedicle screws, transarticular pins and PMMA.^22^ Postoperatively, antibiotics and analgesics were routinely prescribed, most commonly amoxicillin–clavulanic acid and cephalexin. Overall, there is fair evidence supporting decompression with or without stabilisation, particularly in cases of vertebral instability and spinal cord compression. This approach facilitates pathogen identification, culture‐guided antimicrobial selection and may improve long‐term outcomes.

### Discectomy

Three studies reported discectomy as a therapeutic option. One retrospective observational study described percutaneous discectomy performed as a sole treatment working as an intervertebral disc culture,^14^ while two case reports combined discectomy with stabilisation using pedicle screws and PMMA, with one case additionally employing cross‐pinning.^22^, ^40^ All studies showed resolution of DS, short term,^14^ mid term^40^ and long term.^22^ In total, 12 dogs were treated, six of which presented with neurological deficits. Although these studies were classified as moderate‐to‐high risk of bias, disease characterisation was well defined. Fluoroscopy‐guided discectomy may be considered a viable minimally invasive option in cases without vertebral instability, as it allows for decompression and improved pathogen identification.^14^ For study purposes, Kinzel's study was placed in the medical management group.

### Euthanasia

Five studies reported euthanasia without therapeutic intervention^29^, ^30^, ^31^, ^32^, ^33^ (Table 4). All were case reports, including in total five dogs. Three studies identified fungal pathogens, most commonly *Aspergillus* spp.,^29^, ^33^ while the remaining two identified bacterial agents.^30^, ^31^ Disease characterisation was adequate in three studies and poor in two, but all were assessed as high risk of bias, euthanasia was performed either due to the anticipated poor prognosis or the rarity of the causative pathogen in certain geographical regions. These cases highlight the variability in clinical decision making but provide no reliable evidence of treatment efficacy.

**TABLE 4:** Study design, group size, risk of bias and disease characterisation of cases in which euthanasia was performed without attempting any treatment.

References	Study design	Group size	Risk of bias	Disease characterisation
Berry and Leisewitz^29^ ^,a^	Case series (1/2)	Very small	High	Well
Devecioğlu et al.^30^	Case report	Very small	High	Well
Hugnet et al.^32^	Case report	Very small	High	Poor
Pacurar et al.^31^	Case report	Very small	High	Well
Zhang et al.^33^	Case report	Very small	High	Poor

^a^ This study included two dogs, one of which received surgical treatment and was included in group 2, while the other was included in group 3, as euthanasia was performed without any attempted treatment.

## DISCUSSION

To the authors’ knowledge, this is the first systematic review assessing the treatment outcomes of DS in dogs, specifically comparing surgical with medical methods. The review was conducted in accordance with the PRISMA statement guidelines,^59^ and the methodology was designed using the ‘PICO and Search Strategy Worksheet’. A total of 48 studies were included, all classified in group B, as no blinded randomised controlled trials (bRCTs), non‐blinded randomised controlled trials (nbRCTs) or non‐randomised controlled trials (nRCTs) were identified. All selected studies were published in English and collectively included 699 dogs. Of these, 32 studies reported on medical management, while 16 described surgical interventions. Five studies evaluated both surgical and medical approaches. Most studies (68,75%) that employed medical treatment alone reported successful resolution of DS, with antibiotics being the primary modality. Surgical intervention demonstrated a success rate of 75%, predominantly in dogs with neurological deficits. In 33% of the studies, affected dogs did not survive, and in five reports, euthanasia was elected without any attempt of treatment.

The overall risk of bias ranged from moderate/high to high, as no studies were classified under group A. Seven studies in total were classified as moderate/high risk of bias,^3^, ^14^, ^21^, ^22^, ^27^, ^34^, ^40^ while the remainder were considered high risk. The most common sources of bias included retrospective study design, incomplete treatment or follow‐up data, heterogeneity in diagnostic and therapeutic approaches, and short study duration (<6 months). Conflict of interest was disclosed in one study,^6^ and financial support was reported in three.^5^, ^6^, ^23^ Accordingly, the results regarding DS treatment outcomes must be interpreted with caution.

Well‐characterised study groups were present in 35% of the included studies, although only two studies evaluated sufficient case numbers, classified as moderate^27^ and good^10^ group sizes. Both of these involved medical treatments, and the patients achieved resolution of DS in the short term to mid term. However, the healing scale used was greater than 3, which is not considered reliable for confirming resolution. In most studies, a healing scale of 3 was reported, indicating that radiographs were the primary diagnostic tool for assessing resolution. The remaining well‐defined studies evaluated either small or very small groups. Consequently, due to the limited number of well‐characterised studies with adequate group size and the absence of studies in group A, definitive conclusions regarding DS outcome cannot be drawn. Furthermore, neurological status (grade 0–V) could not be analysed, as the majority of the studies failed to provide accurate descriptions of the clinical signs at follow‐up.

Antibiotics were the most frequently reported medical treatment for DS, with several studies showing favourable outcomes. The most commonly prescribed antibiotics were amoxicillin‒clavulanic acid, cephalexin and enrofloxacin. It is noteworthy that basing antibiotic selection on sensitivity tests demonstrates the highest success rate, emphasising the importance of pathogen identification and susceptibility testing prior to initiating treatment. The mean duration of antibiotics was 105 days. All group 1 studies with larger sample size reported successful resolution. Long‐term resolution of DS (>12 months) was achieved in four studies,^9^, ^19^, ^28^, ^34^ while four studies reported short‐term resolution (<6 months)^10^, ^23^, ^35^, ^36^ and one study reported mid‐term resolution (6‒12 months).^27^

Nevertheless, medical management outcomes were highly variable, with failures frequently reported in cases of fungal infection. Although the number of fungal cases was small, the findings suggest a poorer prognosis compared to bacterial DS. Adjunctive medications, such as analgesics, were commonly employed to improve the quality of life of the patients and should be considered in the therapeutic planning. However, corticosteroid administration must be approached with caution, as it has been associated with neurological deterioration.^9^

Overall, medical therapy should be regarded as the first‐line treatment for DS, particularly in the absence of spinal cord compression or vertebral instability. As DS often arises secondary to systemic infection via haematogenous spread, prognosis may be affected by concurrent disease. This review deliberately excluded studies in which DS was secondary to another condition or where patients were critically unstable to specifically assess DS treatment outcomes. However, the variability in medical treatment success, especially in cases involving fungal pathogens or advanced disease, highlights the importance of early diagnosis and close clinical monitoring.

Surgical intervention was typically performed in dogs with severe neurological deficits, with vertebral instability, spinal cord compression identified via advanced imaging, or failure of medical treatment. The most frequently employed techniques included decompression and/or distraction–stabilisation. Surgical management demonstrated favourable outcomes across short‐term,^14^, ^23^, ^52^ mid‐term^27^, ^40^, ^47^ or long‐term^16^, ^19^, ^21^, ^22^, ^28^, ^53^, ^54^ outcomes. This technique demonstrated significant benefits in cases where compression of the spinal cord or vertebral instability was present. Surgery also allowed histopathological and microbiological diagnosis through biopsy, enabling more targeted antibiotic therapy. Therefore, there were more well‐characterised studies of group 2 than of group 1. It is notable that most studies across both groups employed similar antibiotics. In human medicine, treatment in clinically stable patients without pathogen identification is often delayed until causative organisms are isolated by blood culture (multiple samples), surgical biopsy or CT/fluoroscopy‐guided tissue biopsy.^60^

The publication period of included studies may have influenced both diagnostic methods (e.g., advanced imaging and fluoroscopy) and treatment choices, considering the availability of newer medications with fewer side effects in recent years. Historically, radiographs were the primary diagnostic modality, although it is established that there can be a delay of 2‒8 weeks between the onset of clinical symptoms and radiographic signs.^8^, ^34^ Advanced imaging of the epidural space and paravertebral soft tissues is therefore recommended in suspected DS cases to avoid misdiagnosis.^12^ MRI should be considered the gold standard, as CT may fail to detect lesions subsequently identified on MRI.^9^ Some studies relied solely on urine cultures, which have low sensitivity,^19^ highlighting the importance of more accurate diagnostics such as biopsy and fine‐needle aspiration of the IVD. CSF analysis and culture were performed in 10 studies but yielded positive results only occasionally, while blood and tissue cultures were more frequently diagnostic. Tissue culture, obtained by surgical or percutaneous biopsy, remains the most accurate method for pathogen identification. Additionally, C‐reactive protein represents a sensitive though non‐specific biomarker that may have value in diagnosis and follow‐up.^23^

Several factors may influence both diagnostic and treatment of DS. Heterogeneity in diagnostic approaches and therapeutic protocols was a major reason a meta‐analysis could not be performed. Previous trauma or prior steroid treatment has been associated with relapse and neurological deterioration in dogs.^9^ Moreover, dogs with pre‐existing dental disease were reportedly more likely to undergo surgery.^9^ Other factors associated with culture positivity include higher body weight, multiple sampling and institutional differences.^10^ DS can occur at any age,^9^ including in very young dogs,^47^, ^61^ while males appear to be more frequently affected.^4^ Geographic variations in treatment protocols may also exist, particularly for zoonotic agents such as *B. canis*.^62^

This review highlights that both medical and surgical approaches may be effective for DS, depending on disease severity, aetiology and comorbid conditions. Medical treatment appears most successful in cases with well‐defined bacteria aetiology disease, no spinal compression and no vertebral instability. Antibiotic therapy, particularly when guided by culture and sensitivity testing, was associated with improved outcomes. The average duration of medical treatment in this review was 105 days, notably shorter than in a larger excluded study of 516 dogs, which reported an average of 395 days.^4^ This study was excluded in stage 2 due to lack of follow‐up data. Surgical management remains essential for advanced cases, involving vertebral instability, compression or failed medical treatment, with an overall success rate of approximately 75%. Nevertheless, the high risk of bias in many studies indicates potential inflation of success rates and underlines the need for higher quality prospective research. In cats, most of the cases are treated medically,^63^, ^64^, ^65^ with surgical intervention rarely reported.^66^, ^67^ In human medicine, ‘DS’ or ‘spondylodiscitis’ is typically managed medically with pathogen‐directed antibiotics for approximately 6 weeks, guided by culture and sensitivity results.^60^

This review has several limitations that should be acknowledged. Most included studies were judged to be at high risk of bias, largely due to retrospective design and absence of randomisation, thereby reducing evidence quality. Substantial heterogeneity in study design, diagnostic methods, treatment protocols and outcome measures further limits definitive conclusions. Differences in baseline characteristics of the included dogs (e.g., age, breed and sex) precluded a meta‐analytic approach. The small number of studies on antifungal and antiparasitic treatments also restricted evaluation of outcomes for non‐bacterial DS. In addition, the surgical case series is considerably smaller than its medical case counterparts. Furthermore, the frequent inclusion of case reports and very small samples diminishes the reliability of the findings.

## CONCLUSION

Medical management with antibiotics should remain the first‐line treatment of DS, having a 68.75% successful rate, particularly in cases without significant neurological deficits or indications for surgery. Culture and sensitivity testing should be prioritised to optimise antibiotic selection. Further research is required to determine the optimal medical treatment duration, although current recommendations suggest a minimum of 6‒8 weeks. Surgical interventions, with a 75% successful rate, should be considered in cases of medical treatment failure, vertebral instability, or spinal cord compression. Treatment decisions should be guided by advanced imaging and culture results. The potential of minimally invasive surgical techniques, such as percutaneous discectomy, appears promising but warrants further investigation. Overall, the lack of randomised and prospective studies, variations in baseline characteristics of the dog, various sources of bias and the differences in study design all contribute to the low quality of evidence. Therefore, this systematic review underlines the urgent need for well‐designed prospective studies to better define optimal treatment strategies and long‐term outcomes for DS in dogs.

## AUTHOR CONTRIBUTIONS

Vasileios Ioannis Vallios, Holger A. Volk and Marios Charalambous participated in the study design. Vasileios Ioannis Vallios collected and assessed the data for the study with the assistance of Marios Charalambous, Evdokia Sourla, Holger A. Volk and Daniel Low. All the authors helped to draft the manuscript and read and approved the final version.

## CONFLICT OF INTEREST STATEMENT

The authors declare they have no conflicts of interest.

## FUNDING INFORMATION

The authors received no specific funding for this study.

## ETHICS STATEMENT

This systematic review was conducted using data from previously published studies. No new animals experiments, clinical interventions or data collections involving animals or owners were performed. Therefore, ethical approval was not required.

## Data Availability

The datasets analysed during the current study are available from the corresponding author upon reasonable request.
